# In and out among the Hospitals

**Published:** 1888-06-09

**Authors:** 


### In and Out Among the Hospitals.

By a Secretary of Ten Years' Standing.

Copies of Annual Reports and Rules, Accounts of Meetings, Changes in the Staff, &c., should be addressed The Editor, The Lodge, Porchester Square, W.

### PENNSYLVANIA HOSPITAL, UNITED STATES AMERICA.

The Board of Managers of the University of Pennsylvania have just published their annual report. Calculating the dollar at 4*s*. 2*d*., the total receipts for the year have been £9,895 13*s*. 8½*d*., and the expenditure £10,249 6*s*. The daily average number of beds occupied was eighty, and this, after deducting 1*s*. for each out-patient, gives the cost per bed occupied at £71 1*s*. 4*d*., an amount which is very much about the same as an average London hospital. The charity is fairly well endowed, more than half the income being derived from invested property, while a quarter of the expenditure comes from patients' payments, and the rest from voluntary contributions. The pay system adopted is that where every patient pays according to his means. One of the principal features in connexion with the Pennsylvania Hospital is its training school and nurses' home. This is situated in a separate building, and is partly dependent on its own endowments; each nurse has a separate room to herself, and there is also provided a good library and a cheerful sitting and dining room. Every facility is afforded for enabling the nurses to gain a thorough insight into the nature of their work, lectures and demonstrations being given by the visiting and resident staff of the hospital. The Board of Managers consists of the Provost of the University, seven members elected by the subscribers, five by the Board of Trustees, three representatives of the faculty, and three members of the staff elected by the students. Besides this there is a Board of Women Visitors, consisting of twenty-three members, who read to the patients, inspect the hospital, and report to the Board of Managers.

### THE MIDDLESEX HOSPITAL.

There is much to record in connexion with the working of this ancient corporation, and many changes have occurred during the past twelve months. The income has unfortunately been far below the expenditure, the total receipts, including legacies, being £17,919 17*s*. 4*d*., and the expenditure £26,758 17*s*. 8*d*. This latter item includes £5,224 1*s*. 11*d*. extraordinary expenses connected with the almost entire repainting of the building, new cooking apparatus, and other structural alterations and improvements. The Board have wisely adopted the system of cooking by gas, thereby avoiding much expense, besides accomplishing the work of the kitchen in a more orderly and cleanly manner. After deducting for out-patients and extras, the cost per bed occupied was £83 16*s*. 3*d*., and the daily average number of patients 238; this is making allowance for the ten weeks during which the hospital was closed to general cases, and only 35 cancer patients retained. It was very necessary to close the hospital, nearly ten years having elapsed since the premises were last placed in thorough repair. The Board took this opportunity of recording its indebtedness to the authorities of St. Mary's Hospital, and especially to Dr. Cockey, the medical superintendent of that institution, for the assistance rendered in taking over a large number of cases that could not be retained in the wards at the time the hospital was closed. A concert, under the patronage of the Queen, was given at the Royal Albert Hall; it was directed by Sir Arthur Sullivan, and the proceeds amounted to the handsome sum of £559 9*s*. 7*d*. The employés of Messrs. Marshall and Snelgrove also handed over £50 as the proceeds of an entertainment given on behalf of the hospital. Among the personal changes which have taken place during the year may be mentioned the transference of the electrical department, which Dr. Pringle's increasing duties had compelled him to relinquish, to the charge of Dr. Pasteur (the medical registrar). The title of Surgeon to the Hospital has been conferred on Mr. Andrew Clarke, in recognition of the valuable services he has rendered during the long time he has held the office of Assistant Surgeon and Dean of the Medical School. Two vacancies in the Board have been filled up by the election of Mr. Frank Davis and Mr. S. B. Bancroft, both of whom have given, in various ways, abundant proof of their sincere interest in the welfare of the charity. Mr. Lawson and Dr. Powell have been appointed, the former surgeon oculist and the latter physician extraordinary to her Majesty the Queen. Mr. A. O'Donnel Bartholeyns, who had held the appointment of secretary-superintendent since 1885, tendered his resignation, which was accepted by the Board, and on February 21st Mr. Frederick Clare Melhado, who has served for upwards of nine years as assistant secretary, was elected to the vacant office. The Trained Nurses' Institute is now occupying the new buildings adjoining the hospital. Since its establishment the services of the trained nurses have been constantly in demand, and the success which has been attained is principally due to Miss Steuart, whose zeal and activity as lady superintendent is fully recognised. It is pointed out that the institute will besides benefit the hospital by inducing nurses and probationers to remain longer in the service of the hospital, as no nurses are received into the institute until they have served full five years in the wards. Moreover, nurses when not engaged with outside cases are available for service in the hospital. The new residential college forms a conspicuous feature in connexion with the hospital. The buildings are most commodious and substantial, and reflect the greatest credit on Mr. Keith D. Young, the architect. The medical school is to be congratulated on the increasing number of students who are availing themselves of the advantages which the college offers. Mr. Leopold Hudson has been appointed warden. The new buildings of the medical school will benefit not only the students, but the lecturers and staff generally. The new premises comprise a lecture theatre, to seat sixty students, dean's room, students' room, spacious library, physiological laboratory, class rooms, luncheon rooms, &c. It would greatly improve what is an excellent hospital report if a balance-sheet were published as well as the table of income and expenditure.

### ITALIAN HOSPITAL.

All lovers of Italy and the Italian people will take an interest in this hospital. It is managed and supported almost entirely by Italians, and the majority of the patients are their countrymen. The hospital would accommodate 24 patients, but so far the funds at the disposal of the committee have not enabled them to maintain more than 15 beds. The daily average number last year was 11·2. Sir Joseph Fayrer and Sir William Maccormac are consulting physician and surgeon, and are also members of the committee of management. The nursing of the hospital is done by the Sisters of St. Vincent de Paul, whose services are given gratuitously, the charity thus being saved considerable expense. Last year was financially successful, the amonnt received from donations having greatly increased. The total receipts were £947 14*s*. 11*d*., and the expenditure £795 5*s*. 4*d*., after deducting one shilling for each out-patient the cost per bed occupied is £60 16*s*. 1*d*. The hospital is entirely free, the only proviso being that as far as possible preference shall be given to Italians and Italian-speaking people. The report is published in two languages, and is both accurately and carefully compiled.

